# Supplementary material for: SimpactCyan 1.0: An Open-source Simulator for Individual-Based Models in HIV Epidemiology with R and Python Interfaces
Source: Sci Rep. 2019 Dec 17;9:19289. doi: 10.1038/s41598-019-55689-4 (PMC6917719; doi:10.1038/s41598-019-55689-4)
Supplement: Supplementary file 1 — SimpactCyan 1.0: An Open-source Simulator for Individual-Based Models in HIV Epidemiology with R and Python Interfaces [file 41598_2019_55689_MOESM1_ESM.pdf]

SimpactCyan 1.0: An Open-source Simulator for Individual-Based Models in HIV Epidemiology with R and Python Interfaces

Jori Liesenborgs, Diana M Hendrickx, Elise Kuylen, David Niyukuri, Niel Hens, Wim Delva

Table S1. Model calibration results: model and target features.

	Model 1	Model 2	Model 3	Target feature	Source
Demography					World Bank, 2018
Average annual population growth rate in 2000 - 2016	-0.20%	1.10%	2.20%	1.50%	
HIV prevalence					Bicego et al. 2013
18-19 year-old women	18.30%	17.00%	23.10%	14.30%	
18-19 year-old men	1.40%	1.20%	3.40%	0.80%	
20-24 year-old women	29.20%	30.10%	32.80%	31.50%	
20-24 year-old men	7.50%	7.90%	10.30%	6.60%	
25-29 year-old women	47.00%	44.80%	41.90%	46.70%	
25-29 year-old men	21.40%	21.70%	23.80%	21.30%	
30-34 year-old women	52.70%	55.50%	44.60%	53.80%	
30-34 year-old men	35.50%	36.90%	38.30%	36.60%	
35-39 year-old women	50.80%	50.80%	41.80%	49.10%	
35-39 year-old men	38.50%	42.40%	42.50%	47.00%	
40-44 year-old women	45.00%	46.70%	42.40%	39.70%	
40-44 year-old men	37.90%	41.80%	41.70%	45.50%	
45-49 year-old women	39.70%	43.00%	33.40%	31.60%	
45-49 year-old men	33.60%	36.40%	32.40%	42.50%	
HIV incidence (per 100 person-years)					Justman et al. 2017
18-19 year-old women	6.3	5.2	6	3.8	
18-19 year-old men	0.8	1	1.9	0.8	
20-24 year-old women	5.2	5	4.7	4.3	
20-24 year-old men	2.6	2.8	2.9	1.6	
25-29 year-old women	2.4	3.2	2.8	2	
25-29 year-old men	4.7	4.3	4.8	2.6	
30-34 year-old women	1.5	5.6	2.2	2.7	
30-34 year-old men	2.9	4.7	5.4	3.1	
35-39 year-old women	1.3	0.7	0.9	4	
35-39 year-old men	2.5	3.4	2.5	0.4	
40-44 year-old women	0.8	0.2	0.6	2.1	
40-44 year-old men	1.4	1.7	2.8	1.2	
45-49 year-old women	0	0.7	0.3	1.2	
45-49 year-old men	0	1.4	1.3	0	
ART coverage in >=15 year-old adults					UNAIDS, 2019
2010	39.90%	34.20%	25.00%	37%	

2011	46.10%	40.30%	32.00%	40%	
2012	49.40%	43.60%	36.60%	44%	
2013	51.30%	46.00%	40.20%	49%	
2014	64.30%	62.20%	58.50%	58%	
2015	64.80%	64.30%	62.10%	67%	
2016	65.10%	66.20%	64.20%	76%	
2017	78.90%	83.10%	84.20%	85%	
Virology					UNAIDS, 2019
fraction of >=15 year-old adults who were virally suppressed (less than 1000 viral copies per mL blood)	82.50%	85.80%	86.80%	74%	
HIV prevalence in 15-49 year-old adults					UNAIDS, 2019
1990	2.20%	1.70%	1.90%	1.70%	
1991	3.90%	2.40%	3.10%	3.30%	
1992	6.10%	3.80%	5.00%	5.70%	
1993	8.60%	5.60%	7.90%	8.80%	
1994	11.80%	7.90%	11.50%	12.40%	
1995	15.40%	11.10%	15.40%	16.10%	
1996	18.90%	13.90%	18.60%	19.40%	
1997	21.90%	16.40%	20.60%	22.00%	
1998	24.50%	18.90%	22.00%	23.90%	
1999	26.20%	20.80%	22.60%	25.10%	
2000	27.10%	22.00%	22.90%	25.80%	
2001	27.60%	22.80%	23.00%	26.10%	
2002	28.10%	23.50%	22.90%	26.10%	
2003	28.10%	24.20%	22.50%	25.90%	
2004	27.90%	24.50%	22.40%	25.70%	
2005	27.70%	24.80%	22.30%	25.50%	
2006	27.40%	24.80%	22.20%	25.60%	
2007	27.30%	24.80%	22.60%	25.90%	
2008	26.80%	25.00%	22.80%	26.30%	
2009	26.90%	25.30%	23.20%	26.80%	
2010	26.70%	25.40%	23.70%	27.40%	
2011	26.30%	25.40%	24.20%	27.80%	
2012	26.10%	25.40%	24.60%	28.20%	
2013	26.20%	25.50%	25.00%	28.40%	
2014	25.90%	25.50%	25.00%	28.40%	
2015	25.70%	25.20%	25.00%	28.30%	
2016	25.50%	25.00%	24.90%	27.90%	
2017	25.10%	24.40%	24.50%	27.40%	

**Table S2. Evolutionary models fitted to empirical and synthetic data.**		
	**Empirical data**	**Synthetic data**
Relative Frequencies		
adenine (A)	0.3906	0.3929
cytosine (C)	0.1752	0.1726
guanine (G)	0.2201	0.2234
thymine (T)	0.2142	0.2111
Rate heterogeneity		
shape parameter	0.6250	0.6244
Relative substitution rates		
r(A → G) = r(G → A)	1.9803	2.0421
r(A → C) = r(C → A)	9.4404	9.5318
r(A → T) = r(T → A)	0.9423	0.9674
r(G → C) = r(C → G)	0.8770	0.8840
r(G → T) = r(T → G)	11.6367	12.0604
r(C → T) = r(T → C)	1.0000	1.0000
Fraction of invariant sites		
I parameter	0.2130	0.3091

Table S3. Normalised topological properties of the phylogenetic trees reconstructed from empirical and synthetic data with matching sampling dates (See also Figure S1).

	**Empirical data**	**Synthetic data**
Sackin index of tree imbalance	0.1020	0.0880
Colless index of tree imbalance	0.0760	0.0590
Average size of ladders[1]	0.0060	0.0070
Cherries[2]	0.6630	0.6350
IL number[3]	0.3390	0.3660
Maximum height of the tips	0.0830	0.0730
Pitchforks[4]	0.4510	0.4770
First staircase-ness measure[5]	0.6000	0.6320
Second staircase-ness measure[6]	0.6030	0.5980

1 A ladder is defined as a series of consecutive nodes in the tree, each of which has exactly one tip child. The size of the ladder is given by the number of nodes in the chain.

2 A cherry is a pair of sister tips.

3 The IL number is defined as the number of internal nodes with a single tip child.

4 Pitchforks are clades with three tips.

5 The proportion of subtrees that are imbalanced (i.e. subtrees where the left child has more tip descendants than the right child, or vice versa).

6 The average of all the min(l,r)/max(l,r) values of each subtree, where l and r are the number of tips in the left and right children of a subtree.

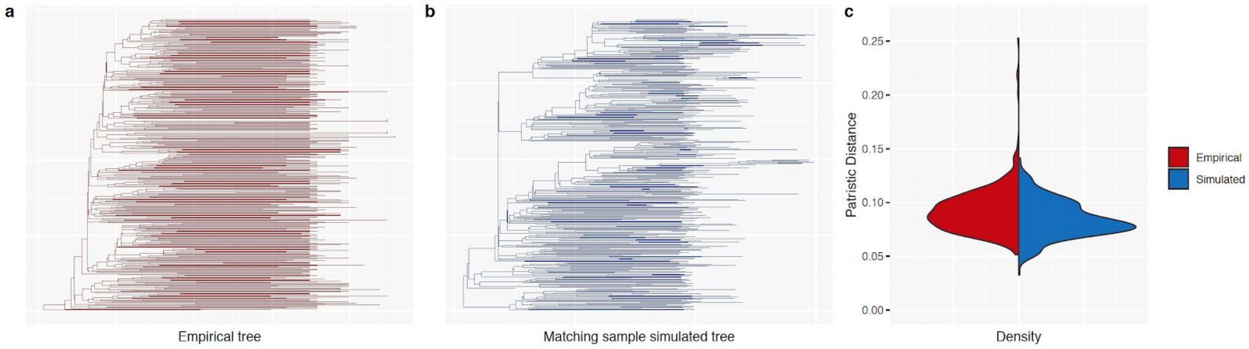

Figure S1. (**a**) Time-resolved phylogenetic tree, reconstructed from the empirical HIV sequence data. (**b**) Time-resolved phylogenetic tree, reconstructed from a subset of the synthetic HIV sequence data, with sampling dates that match those of the empirical dataset. (**c**) The density distribution of patristic distances of the respective phylogenetic trees.
